# Diversity and overlap of parvalbumin and somatostatin expressing interneurons in mouse presubiculum

**DOI:** 10.3389/fncir.2015.00020

**Published:** 2015-05-08

**Authors:** Mérie Nassar, Jean Simonnet, Roxanne Lofredi, Ivan Cohen, Etienne Savary, Yuchio Yanagawa, Richard Miles, Desdemona Fricker

**Affiliations:** ^1^Institut du Cerveau et de la Moelle Epinière, Sorbonne Universités, UPMC Université Paris 06 UM 75, CHU Pitié-Salpêtrière INSERM U1127, CNRS UMR7225Paris, France; ^2^Neuroscience Paris Seine Paris, Sorbonne Universités, UPMC Université Paris 06 UM CR 18, CNRS UMR 8246, INSERM U1130Paris, France; ^3^Department of Genetic and Behavioral Neuroscience, Gunma University Graduate School of MedicineMaebashi, Japan; ^4^Japan Science and Technology AgencyTokyo, Japan

**Keywords:** inhibition, excitability, morphology, postsubiculum, head direction

## Abstract

The presubiculum, located between hippocampus and entorhinal cortex, plays a fundamental role in representing spatial information, notably head direction. Little is known about GABAergic interneurons of this region. Here, we used three transgenic mouse lines, Pvalb-Cre, Sst-Cre, and X98, to examine distinct interneurons labeled with tdTomato or green fluorescent protein. The distribution of interneurons in presubicular lamina for each animal line was compared to that in the GAD67-GFP knock-in animal line. Labeling was specific in the Pvalb-Cre line with 87% of labeled interneurons immunopositive for parvalbumin (PV). Immunostaining for somatostatin (SOM) revealed good specificity in the X98 line with 89% of fluorescent cells, but a lesser specificity in Sst-Cre animals where only 71% of labeled cells were immunopositive. A minority of ∼6% of interneurons co-expressed PV and SOM in the presubiculum of Sst-Cre animals. The electrophysiological and morphological properties of fluorescent interneurons from Pvalb-Cre, Sst-Cre, and X98 mice differed. Distinct physiological groups of presubicular interneurons were resolved by unsupervised cluster analysis of parameters describing passive properties, firing patterns and AP shapes. One group consisted of SOM-positive, Martinotti type neurons with a low firing threshold (cluster 1). Fast spiking basket cells, mainly from the Pvalb-Cre line, formed a distinct group (cluster 3). Another group (cluster 2) contained interneurons of intermediate electrical properties and basket-cell like morphologies. These labeled neurons were recorded from both Sst-Cre and Pvalb-Cre animals. Thus, our results reveal a wide variation in anatomical and physiological properties for these interneurons, a real overlap of interneurons immuno-positive for both PV and SOM as well as an off-target recombination in the Sst-Cre line, possibly linked to maternal cre inheritance.

## Introduction

The presubicular cortex, located between the hippocampus and the medial entorhinal cortex, plays a major role in spatial navigation. It contains “head direction” cells which discharge according to the orientation of the animal’s head in the environment (Taube et al., 1990; Van Strien et al., 2009; Boccara et al., 2010). Visual information from visual and retrosplenial cortices, and directional information from vestibular nuclei converge in the presubiculum (Calton et al., 2003; Taube, 2007). Presubicular output neurons project directional information to grid cells in the entorhinal cortex (van Groen and Wyss, 1990a; van Haeften et al., 1997; Honda and Ishizuka, 2004; Yoder et al., 2011; Kononenko and Witter, 2012; Rowland et al., 2013).

Three types of pyramidal cells have been distinguished in superficial and deep layers of presubiculum (Simonnet et al., 2013). They all receive frequent inhibitory synaptic events. Spatial information processing in the presubiculum involves local interactions between excitatory glutamatergic neurons and inhibitory GABAergic interneurons. The physiological and anatomical features of presubicular interneurons and their distribution in superficial and deep layers are not yet well defined. In hippocampus and neocortex, distinct subsets of interneurons are believed to play distinct roles. In particular, soma targeting inhibitory neurons control timing and frequency of AP discharge in pyramidal cells (Miles et al., 1996; Fricker and Miles, 2001) and may contribute to the generation of fast oscillations (Cobb et al., 1995; Chrobak and Buzsaki, 1996; Somogyi and Klausberger, 2005; Schlingloff et al., 2014). Dendrite targeting interneurons rather control input signals to pyramidal neurons (Isaacson and Scanziani, 2011). Do analogous interneuron types with comparable functions contribute to represent head direction in the presubicular microcircuit?

Different interneuron functions are mediated by heterogeneous GABAergic cells of multiple embryonic sources (Kepecs and Fishell, 2014). Interneurons may be subdivided according to their somato-dendritic form, synaptic connectivity, electrophysiology and neurochemistry (Freund and Buzsáki, 1996; Cauli et al., 1997; Kawaguchi and Kubota, 1997; Parra et al., 1998; Markram et al., 2004; Rudy et al., 2010; Defelipe et al., 2013; Kubota, 2014). The definition of an interneuron type is still open to debate. Classification by unsupervised clustering has been used to define neuronal classes through sets of common features (Dumitriu et al., 2006; Ma et al., 2006; Karagiannis et al., 2009; McGarry et al., 2010; Perrenoud et al., 2012; Helm et al., 2013). Even so, Battaglia et al. (2013) note that a continuum of phenotypes may exist.

We therefore characterized the electrophysiology and anatomy of two well-defined groups of presubicular interneurons. Interneurons were recorded in slices from three transgenic mouse lines. In Pvalb-Cre tdTomato animals, cells expressing the calcium binding protein PV should be fluorescent, and neurons expressing the neuropeptide SOM should be labeled in Sst-Cre tdTomato and X98 GFP mice. Unsupervised cluster analysis of physiological parameters revealed three main groups of interneurons. These subsets did not coincide perfectly with neurochemical marker expression. Our results establish a dichotomy between Pvalb and X98 interneurons in presubiculum, while Sst-Cre neurons showed similarities with both the X98 and the Pvalb interneurons, partially depending on the parent-of-origin for cre transmission.

## Materials and Methods

### Animals

Experiments were performed on male and female Pvalb-Cre mice (Jax 008069; Hippenmeyer et al., 2005) and Sst-IRES-Cre mice (Jax 013044; Taniguchi et al., 2011) crossed with the Ai14 Cre reporter line (Jax 007914; Madisen et al., 2010). Cre-mediated recombination resulted in the expression of red fluorescent tdTomato labeling in subsets of GABAergic neurons. The terms “Sst-Cre” or “Pvalb-Cre” will be used when referring to the mouse line. “SOM” and “PV” will be used to refer to the expression of the neuropeptide or Ca-binding protein marker. We will show that these terms are not always equivalent. We also used a transgenic mouse line X98 (Jax 006340), in which GFP expression driven by the GAD67 short promoter, labels a subset of SOM positive neurons. With axons arborizing in layer I, these cells resemble neocortical Martinotti cells (Ma et al., 2006). The total number of interneurons in all presubicular layers was quantified using GAD67-GFP knock-in mice, in which GFP was specifically expressed in GABAergic neurons under the control of the endogenous GAD67 promoter (Tamamaki et al., 2003). Our care and use of animals conformed to the European Community Council Directive of 22 September 2010 (2010/63/EU) and French law (87/848). Our study was approved by the local ethics committee Charles Darwin N°5 and the French Ministry for Research.

### Immunohistochemistry

Mice were anesthetized intraperitoneally with ketamine hydrochloride and xylazine (100 and 15 mg.kg^-1^, respectively). They were then perfused transcardially with 0.9% saline containing heparin (100–200 UI/ml followed by 30–50 ml of a fixative solution containing 4% paraformaldehyde in 0.1 M phosphate buffer (PB). Dissected brains were post-fixed overnight in the same solution at 4°C, rinsed three times for 3 min, and then placed in a 30% sucrose solution at 4°C for at least 24 h. Horizontal sections of thickness 60 μm were cut in 0.1 M PBS using a slicing vibratome (Microm HM650 V). Membranes were permeabilized by three cycles of freeze-thawing slices on dry ice in a 30% sucrose containing solution. Sections were washed three times (2 × 30 min, 1 × 60 min) in PBS 0.1M (BupH^TM^ Phosphate Buffered Saline Packs, Thermo Fisher Scientific), then transferred to a saturation buffer containing 2% milk powder and 0.3–0.4% Triton X-100 in PBS 0.1M, and agitated for 2 h at room temperature. Sections were then transferred into primary antibody solution of 0.1M PBS and 0.3% Triton X-100 and gently agitated, overnight at 4°C. Sections were rinsed three times (2 × 30 min, 1 × 60 min) in PBS then incubated in dilutions of secondary antibody, conjugated to different fluorophores, for 4 h at room temperature under gentle agitation. 4′,6-diamidino-2-phenylindole (DAPI, Sigma) was always added to secondary antibodies containing solutions (1:1000) to stain cellular nuclei. For SOM immunostaining, we increased the incubation time with the primary antibody to 48–72 h and with the secondary antibody to 24 h, both at 4°C. For SOM and PV co-labeling, these long incubation times were applied as well.

The following primary antibodies were used: Goat Anti-PV (Swant, PVG-214, 1:500), Rat Anti-Somatostatin (Chemicon #MAB357, 1:200), Mouse Anti-NeuN (Millipore #MAB377, 1:500), rabbit anti-GFP (Millipore #AB3080, 1:500). Secondary antibodies were: Donkey Anti-Mouse (Cy3, Jackson ImmunoResearch, 1:500), Donkey Anti-Rat (Millipore, A488, 1:500), Donkey Anti-Rabbit (Cy2, Jackson, 1:500), Donkey Anti-Goat (Life technologies A647 or A488, 1:500). Stained sections were mounted on glass slides, coverslipped with anti-fade Prolong Gold (Life technologies).

### Image Acquisition and Analysis

Stained slices were visualized with a QImaging Retiga EXI camera (Qimaging Surrey, BC, Canada), and scanned with an Optigrid II (Thales Optem, Qioptik, Rochester, NY, USA) on an inverted Olympus IX81 microscope. The Optigrid system permitted the acquisition of structured images Stacks of 50–80 images (*z*-step, 0.7 μm) were acquired per slice, using an oil immersion objective (20x, NA 0.9). Presubicular layers and borders were defined using specific cytoarchitectonic features identified by DAPI staining. Images were uniformly adjusted for contrast and brightness.

For each brain slice, fluorescent tdTomato+ or GFP+ cells from all layers of the presubiculum were identified visually by complete scans of optical sections. Cell counting was performed using Volocity software (Improvision, Perkin-Elmer, Coventry, UK) to measure cell numbers in defined volumes. Counts were made only from slices with optimal signal to noise levels and very low background fluorescence. Visual checks only revealed very rare ambiguities for weakly fluorescent neurons. Cell density was calculated as the number of fluorescent cells/volume of each presubicular layer (nb/mm^3^).

Antibody fluorescence was examined for each tdTomato+ or GFP+ cell. A cell was regarded as positive for a given antibody when somatic fluorescence was clearly higher than background levels. Colocalization of antibody labeling was confirmed from observations at all levels of a stack of optical sections for the soma of a neuron. Percentages of single and dual-immuno labeled fluorescent neurons were obtained by dividing the number of immuno-labeled fluorescent neurons by the total number of GFP+ or tdTomato+ neurons. Data are given as mean ± SEM.

### Slice Preparation for Patch-Clamp Recording

Acute slices containing the hippocampus, subicular complex and entorhinal cortex were prepared from 21 to 45 days-old mice. After ketamine hydrochloride and xylazine anesthesia (100 and 15 mg.kg^-1^, respectively), animals were perfused through the heart with a solution containing 125 NaCl, 25 sucrose, 2.5 KCl, 25 NaHCO_3_, 1.25 NaH_2_PO_4_, 2.5 D-glucose, 0.1 CaCl_2_, 7 MgCl_2_ (in mM) cooled to 4°C and equilibrated with 5% CO_2_ in O_2_. Animals were decapitated and horizontal, 280–320 μm thick brain sections were cut in the same solution using a vibratome (Leica VT1000S or Microm HM650V). They were stored for at least 1 h at 22–25° C in a holding chamber filled with ACSF containing (in mM): 124 NaCl, 2.5 KCl, 26 NaHCO_3_, 1 NaH_2_PO_4_, 2 CaCl_2_, 2 MgCl_2_, and 11 D-glucose, bubbled with 5% CO_2_ in O_2_ (pH 7.3, 305–315 mOsm/L). Slices were then transferred to a recording chamber (volume 2–3 ml, temperature 33–35°C) mounted on either an Axioskop 2 FS plus microscope (Zeiss, France) or a BX51WI microscope (Olympus, France).

### Whole-Cell Patch-Clamp Recordings

Recordings were made with glass pipettes pulled using a Brown–Flaming electrode puller (Sutter Instruments) from borosilicate glass of external diameter 1.5 mm (Clark Capillary Glass, Harvard Apparatus). Electrode resistance was 3–6 MΩ after filling with a solution containing (in mM): 135 K-gluconate, 1.2 KCl, 10 HEPES, 0.2 ethylene glycol tetraacetic acid (EGTA), 2 MgCl_2_, 4 MgATP, 0.4 Tris-GTP, 10 Na_2_-phosphocreatine and 2.7–7.1 biocytin. The pH of the pipette solution was adjusted to 7.3 with KOH and the osmolarity was 290 mOsm. Slices were visualized using infrared-differential interference contrast optics. Fluorescently labeled PV, SST or X98 interneurons were identified using LED illumination with appropriate emission/excitation filters (OptoLED, Cairn Research, Faversham, UK). Whole-cell current-clamp recordings were made using a MultiClamp 700B amplifier and pCLAMP software (Molecular Devices, Union City, CA, USA). Potential signals were filtered at 6 kHz and digitized at 20–50 kHz and an estimated junction potential of ∼15 mV was not corrected.

### Electrophysiological Analysis

Recorded signals were analyzed with Axograph X and routines written in MATLAB (The Mathwork). Cellular parameters were measured at least 3–5 min after whole-cell records were established. RMP was the mean potential over at least 10 s. Most electrophysiological parameters were measured from responses to step current injections of 800 ms duration applied from a fixed membrane potential of -65 mV. Injected currents increased from negative to positive values, with a range of amplitudes that resulted in hyperpolarization to about -100 mV during the first step and depolarizations to maximum sustained firing frequency. Depending on the resistance of the recorded neuron, the first hyperpolarizing step was in the range of -50 pA to -500 pA with a step change of 5–50 pA. Neuronal R_in_ was determined as the slope of the current-voltage (I-V) relationship between -71 and -64 mV. Membrane time constants (τ) were estimated from a double exponential fit to the negative deflection of membrane voltage (Levenberg–Marquardt algorithm; Golowasch et al., 2009) in response to a 800 ms hyperpolarizing current injection inducing a voltage change of up to 15 mV. A “sag ratio,” indicative of I_h_ expression, was calculated for steps in which the voltage deflection reached values between -105 and -90 mV, as the ratio of the maximal negative potential, typically from 0 to 200 ms, divided by the mean steady state voltage deflection, between 400 and 800 ms.

Action potentials of amplitude at least 20 mV were detected from continuous periods of rising membrane potential. Rheobase (or threshold current for firing) was defined as the smallest current step of 800 ms that elicited at least one AP. Firing frequency (Hz) was deduced either by averaging all instantaneous frequencies of a given step (MeanInsF) or dividing the number of APs over time (*APs/sec*). Input–output (I–O) curves were constructed by plotting firing frequency (either *MeanInsF* or *APs/sec*) as a function of injected current. The I–O gain was measured from a linear fit to frequencies measured from the nine current steps after rheobase. The coefficient of variation (CV) of firing frequency was calculated as SD divided by the mean of InsF when at least three APs were elicited. This value gave an index of firing regularity; values were higher when firing was more irregular. First AP-Latency was calculated from the first AP in spike trains induced by injecting a current of amplitude twice rheobase. The adaptation index (AI) was defined as the ratio of the mean of the three last instantaneous frequencies divided by the first InsF, measured from a step with minimum CV.

Action potential waveform features were obtained by averaging the measures from the first AP elicited, at latency less than 100 ms, by three consecutive depolarizing steps. AP threshold (threshold) was defined as the membrane potential when dV/dt > 30 mV/ms. AP peak was its maximum potential. The AP rising amplitude (amplitude) was the difference between the threshold and the peak AP voltage. AP width (width) was measured from the half-height of the AP rising phase. *Max depolarization rate* and *max repolarization rate* were defined as the maximum and minimum dV/dt, during rising and falling phases of APs, respectively. The AHP was the voltage minimum after the AP peak and its amplitude (AHP) was defined as the difference from the threshold.

### Cluster Analysis

We performed unsupervised cluster analysis using 17 electrophysiological parameters from 159 neurons recorded in superficial and deep layers of the presubiculum. The parameters were: (1) RMP, (2) R_in_, (3) tau, (4) sag ratio, (5) rheobase, I–O gain ((6) *MeanInsF* or (7) *APs/sec);* (8) *MeanInsF* at 2 times rheobase, (9) CV, (10) latency, (11) AI; AP properties including (12) threshold, (13) width, (14) amplitude, (15) AHP, (16) maximum depolarization rate and (17) maximum repolarization rate.

Interneurons were grouped on similarities of these parameters, using Ward’s method (Ward, 1963), with Euclidean distances measured as previously described (Simonnet et al., 2013). Cluster analysis was implemented using the statistics toolbox of MATLAB (The Mathwork). The Thorndike procedure (Thorndike, 1953), where jumps in distances within clusters indicate prominent differences between groups, was used to examine resulting clusters.

### Neuronal Morphology: Staining, Image Acquisition, and 3D Reconstruction

After recordings with pipettes containing biocytin (1–3 mg.ml^-1^), slices were fixed in 4% paraformaldehyde in 0.1 M PB at 4°C for 24 h. Slices were then rinsed in PBS (3 × 3 min) and cryoprotected in 30% sucrose mixture at 4°C overnight. Membranes were permeabilized by three cycles of freeze-thawing over dry ice and then washed three times with PBS (2 × 30, then 1 × 60 min). Slices were agitated in saturation buffer containing 2% milk powder and 1% Triton X-100 in PBS 0.1M for 3 h at room temperature. Then, section were gently agitated with Streptavidin–Cy3 or Cy5 conjugate (1:500, Invitrogen, Eugene, OR, USA) and DAPI in the blocking solution overnight at 4°C. After washing with PBS (2 × 30 min, 1 × 60 min). Slices were mounted on coverslips using anti-fade Prolong Gold medium (Life technologies). Filled cells were visualized with a QImaging Retiga EXI camera on an inverted Olympus IX81 microscope. Structured images were acquired with an Optigrid system and Volocity software (Improvision, Perkin-Elmer, Coventry, UK). Stacks of 75–250 images (*z*-step 0.7 μm) were acquired with a 20X, 0.9NA oil immersion objective. Stacks were then imported to the Neurolucida software (Microbrightfield, Williston, VT, USA) for three-dimensional reconstruction. The “layer length” analysis feature of the Neurolucida software was used to measure dendritic and axonal lengths in specific layers of the presubiculum as previously (Simonnet et al., 2013). DAPI staining was used to define boundaries and layers of the presubiculum. We did not correct for tissue shrinkage.

### Statistics

Results are given as mean ± SEM. Statistical analysis was performed with Prism (GraphPad Software, Inc.) and MATLAB (The Mathwork). The Wilcoxon signed rank test for matched pairs was used to compare non-parametric data in matched samples. The Kruskal–Wallis one-way analysis of variance (ANOVA) test followed by Dunn’s *post hoc* comparison was used for comparison between more than two groups. Significance levels are indicated as *p* values.

## Results

### Layer Distribution and Immunohistochemistry of GABAergic and non-GABAergic Neurons in Mouse Presubiculum

**FIGURE 1:**
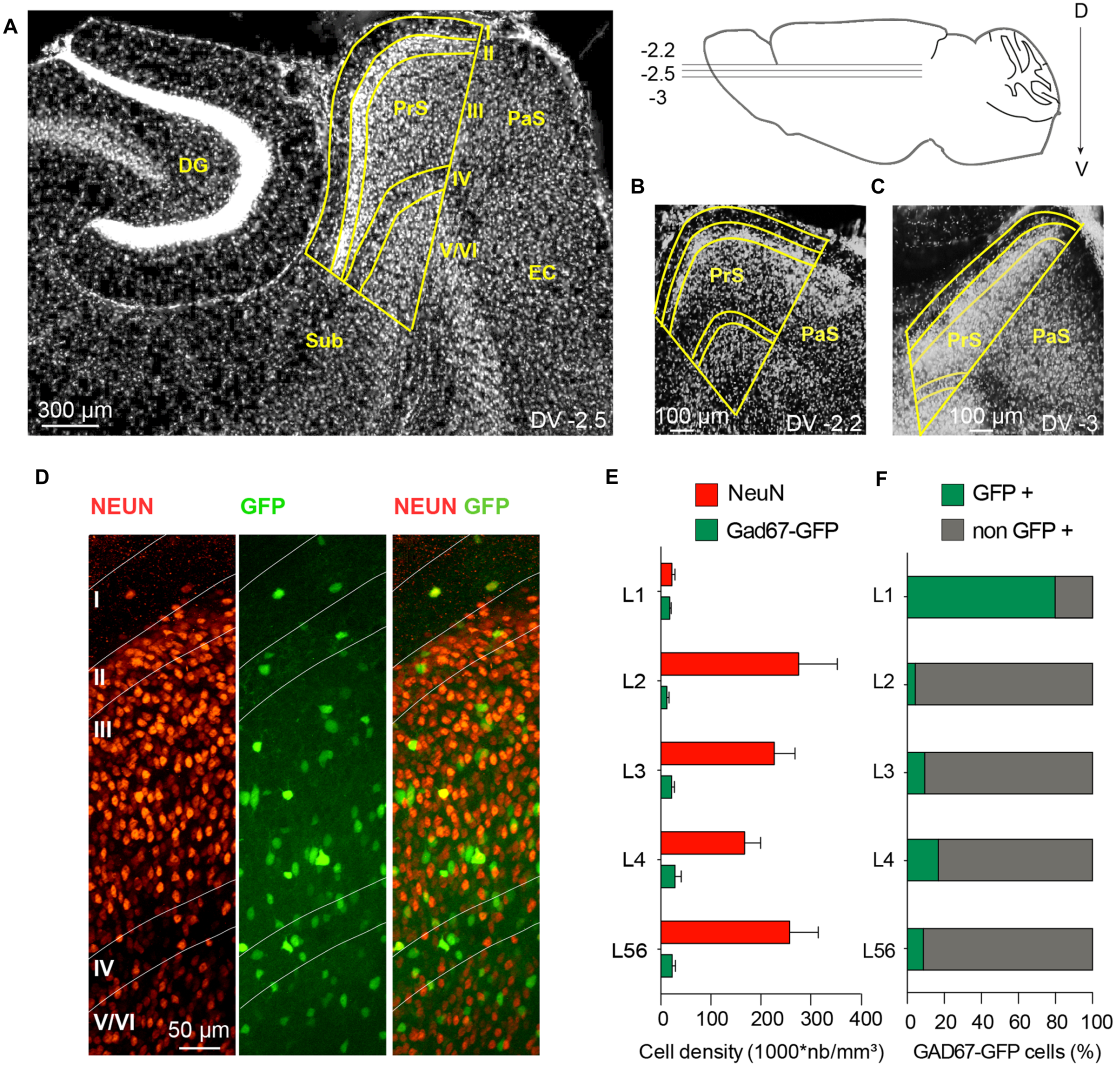
**Spatial distribution of GABAergic and non-GABAergic neuron somata in mouse presubiculum. (A)** Horizontal section of mouse presubiculum (PrS) stained with DAPI. Dorso-ventral (DV) level -2.5 mm. The presubiculum continues from the subiculum (Sub), faces the dentate gyrus (DG), and is adjacent to parasubiculum (PaS) and entorhinal cortex (EC). Changes in the disposition of these regions at DV -2.2 mm **(B)** and DV -3 mm **(C)**. **(D)** NeuN staining (left), GFP fluorescence (middle), and an overlay (right) of all layers of presubiculum in a slice from a GAD67-GFP mouse. **(E)** Mean density of NeuN stained neurons (red) and GAD67-GFP+ neurons (green) for each layer. Mean neuronal density and its standard error were measured from 12 horizontal slices from 3 mice. **(F)** Proportions of GAD67-GFP+ neurons (green) and non-GABAergic neurons (NeuN+, GFP-, gray) in each layer. Scale bars: **(A)** 300 μm; **(B,C)** 100 μm; **(D)** 50 μm.

**Figure 1A** shows the presubiculum in the context of the mouse hippocampal formation. Six cytoarchitectonic layers can be recognized. The high density of cell bodies in layer II serves as a good marker to define the proximal transition to the subiculum and the distal border with parasubiculum. In ventral horizontal sections, the presubiculum is small with a triangular shape; it becomes broader in dorsal sections. Dorsal presubiculum is also termed postsubiculum (van Groen and Wyss, 1990a; Figures 1A–C). Most presubicular neurons are glutamatergic and a smaller proportion are GABAergic. We measured the densities and distributions of GABAergic and non-GABAergic neurons at mid-dorsal level (**Figure 1A**). In 12 slices from 3 adult GAD67-GFP knock-in animals (Figures 1D–F), NeuN labeled neurons and GFP+ neurons were counted in superficial (I, II, and III) and deep layers (IV, V/VI). NeuN labeling was sparse in layer I, contrasting with a high neuronal density in layer II (275 651 ± 134 225 cells/mm^3^). Neuronal density in layer III was apparently lower and that in layer IV even lower (167 484 ± 55 674 cells/mm^3^). Neuronal density in layers V and VI, which are not readily distinguished, increased toward levels similar to those of layer III. We assume that GFP-labeled neurons of the GAD67 knock-in line represent all GABAergic neurons of the presubiculum. Overall, 11% of all neurons were GFP-positive. This ratio is similar to the proportion of interneurons in hippocampus and neocortex. GABAergic cell somata were present in all layers, including layer I. The highest laminar density of about 30 000 GFP+ neurons/mm^3^ was detected in layer IV. GABAergic GFP+ neurons were a majority, ∼80%, of all NeuN labeled neurons in layer I. Lower proportions of GABAergic to NeuN labeled neurons were found in all other layers: 4% in layer II, 9% in layer III, 16% in layer IV and 9% in layers V/VI.

We examined the presubicular distribution of labeled neurons in transgenic mice created to label cells expressing PV or SOM: female Pvalb-Cre (Hippenmeyer et al., 2005) and female Sst-IRES-Cre mice (Taniguchi et al., 2011) were crossed with a reporter line expressing a red fluorescent protein, tdTomato (Ai14, Madisen et al., 2010). We also examined the X98 mouse line (Ma et al., 2006), where a subset of SOM expressing interneurons is labeled with GFP.

**FIGURE 2:**
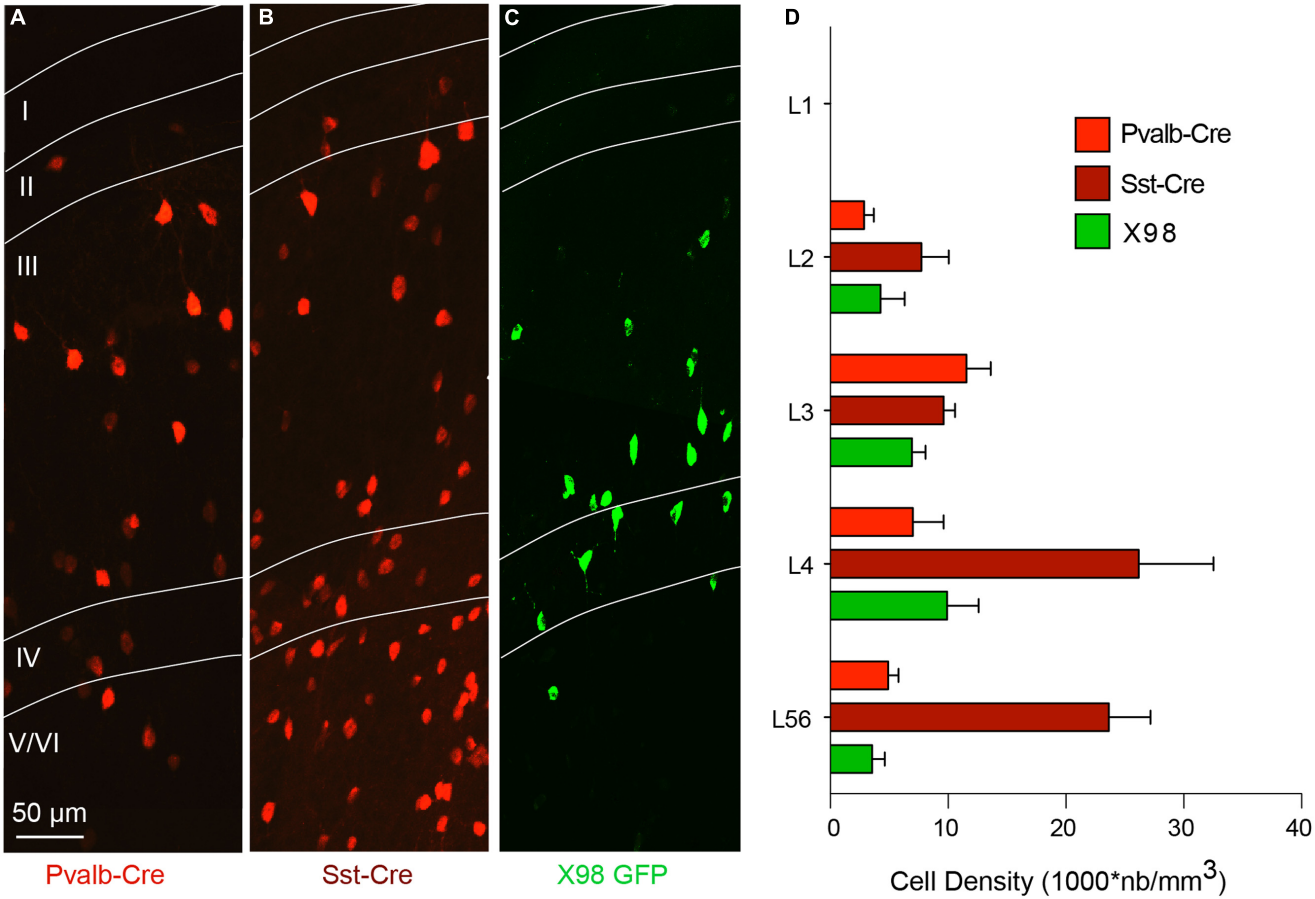
**Layer-specific distribution of presubicular interneurons in three mouse lines. (A–C)** show horizontal sections of presubiculum, with fluorescently labeled neurons from the Pvalb-Cre line **(A)**, the Sst-Cre line **(B)**, both with maternal cre inheritance and tdTomato as reporter (red), and GFP+ interneurons from an X98 animal (green; **C**). Layers are indicated and the scale bar is 50 μm **(D)** Densities (mean ± SEM) of labeled neurons from these three lines in different layers of presubiculum. Data from 6 slices of three mice for each strain.

The distribution of interneurons in these mouse lines was quantified as the density of fluorescently labeled cells, in different layers of the presubiculum, and compared to that of interneurons in GAD67-GFP mice. Pvalb-Cre interneurons were absent from layer I. Their mean density was higher in layer III than in layer II or in layers V/VI (**Figures 2A,D**). Pvalb cell bodies were smaller and neurite staining less intense than in neighboring parasubiculum (not shown). tdTomato-labeled Sst-Cre interneurons were more abundant in deep than in superficial layers, with maximal densities in layer IV and upper layer V/VI (**Figures 2B,D**). No Sst-Cre interneurons were found in layer I. GFP-labeled neurons of the X98 line were sparse, with less cells labeled than in the Pvalb or Sst-Cre line. The density of labeled interneurons was highest in layer IV, and no cells were labeled in layer I (**Figures 2C,D**). We noted that the density of Sst-Cre tdTomato labeled neurons in layers V/VI exceeded that of GAD67-GFP+ neurons. This was unexpected, since all GABAergic neurons should be labeled in GAD67-GFP animals (Tamamaki et al., 2003) and in the Sst-Cre line only a subset of SOM expressing interneurons should be labeled.

**FIGURE 3:**
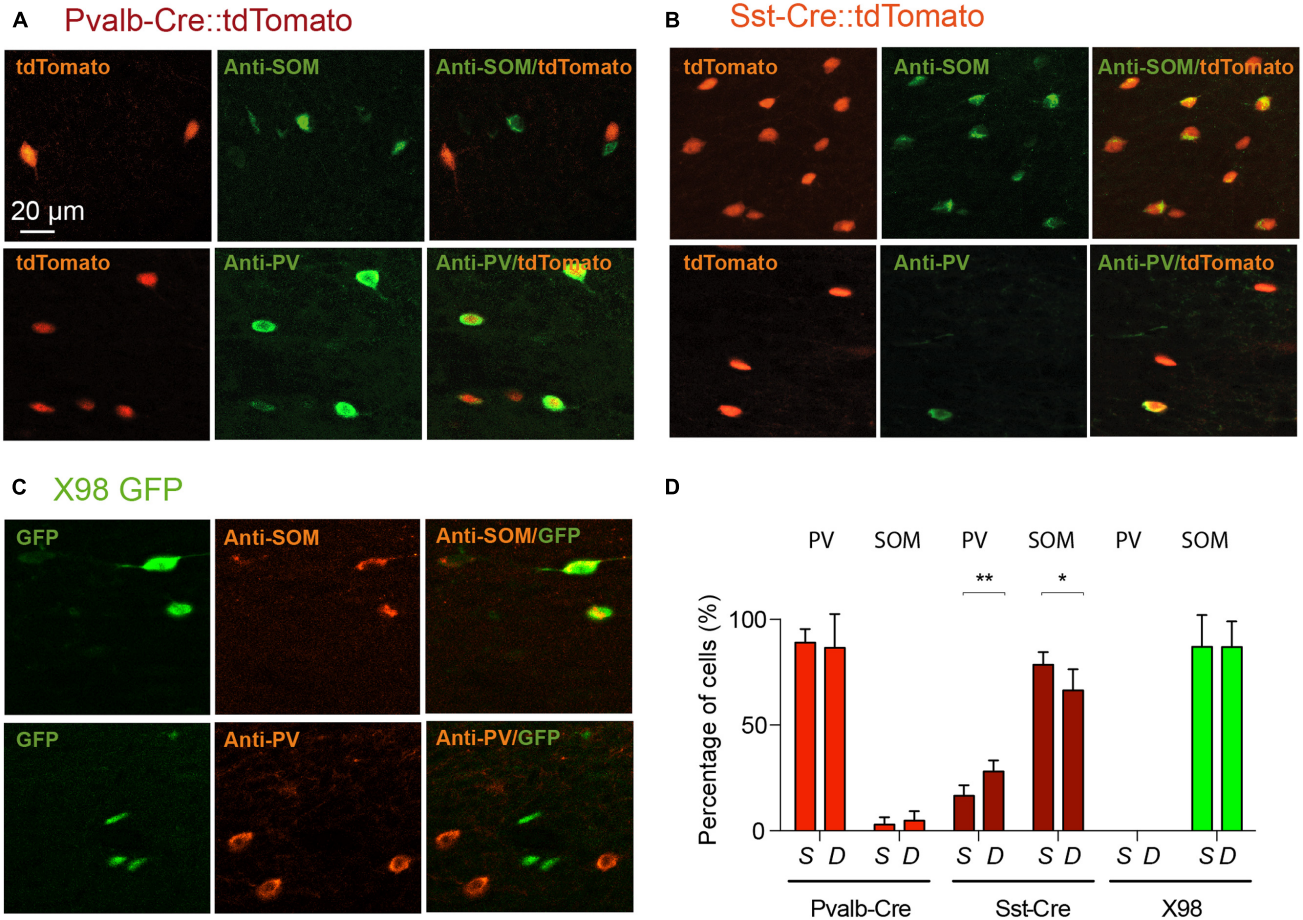
**Verification by PV or SOM immunostaining of the identity of labeled neurons from Pvalb-Cre tdTomato, Sst-Cre tdTomato, or X98 GFP animals**. Presubicular sections from **(A)**, Pvalb-Cre tdTomato mice and **(B)** Sst-Cre tdTomato mice, both with maternal cre inheritance, and **(C)**, X98 GFP mice, immunostained with anti-SOM (top) and anti-PV antibodies (bottom). For each panel fluorescent labeled neurons of the cell line are on the right, immunostaining in the middle and merge on the left. The scale bar is 20 μm. **(D)** Bar graphs indicate percentages of antibody labeling of fluorescent (tdTomato or GFP) neurons (mean ± SEM) from each mouse line. Data from at least 6 slices from 3 animals of each mouse line and each antibody (Wilcoxon’s test, ^∗^*P* < 0.05, ^∗∗^*P* < 0.01).

We therefore used antibodies against PV and SOM to explore the specificity of labeled cells in the Pvalb-Cre and Sst-Cre lines and in X98 mice. The numbers of tdTomato+ or GFP+ fluorescent presubicular cells that were also immunopositive for PV or SOM were quantified in at least three non-adjacent slices from at least three animals for each line (**Figures 3A,D**). As expected, the great majority of tdTomato+ neurons in the Pvalb-Cre line were immunopositive for PV (326/374); very few were positive for SOM (7/374). Also, most GFP+ neurons of the X98 line were positive for SOM (155/175); none were labeled for PV (0/175). However for the Sst-Cre line, while 403 out of 570 tdTomato+ presubicular cells were positive for SOM, 154 out of 674 Sst-Cre tdTomato+ cells were positive for PV (**Figures 3C,D**). The SOM immuno-labeling was significantly lower in deep layers (IV–VI) of presubiculum (66 ± 5%) than in superficial layers (78 ± 3%; Wilcoxon matched-pairs signed rank test, *P* < 0.05), while PV labeling was significantly higher in deep layers (28 ± 3%) than in superficial layers (16 ± 3%; Wilcoxon matched-pairs signed rank test, *P* < 0.01).

**FIGURE 4:**
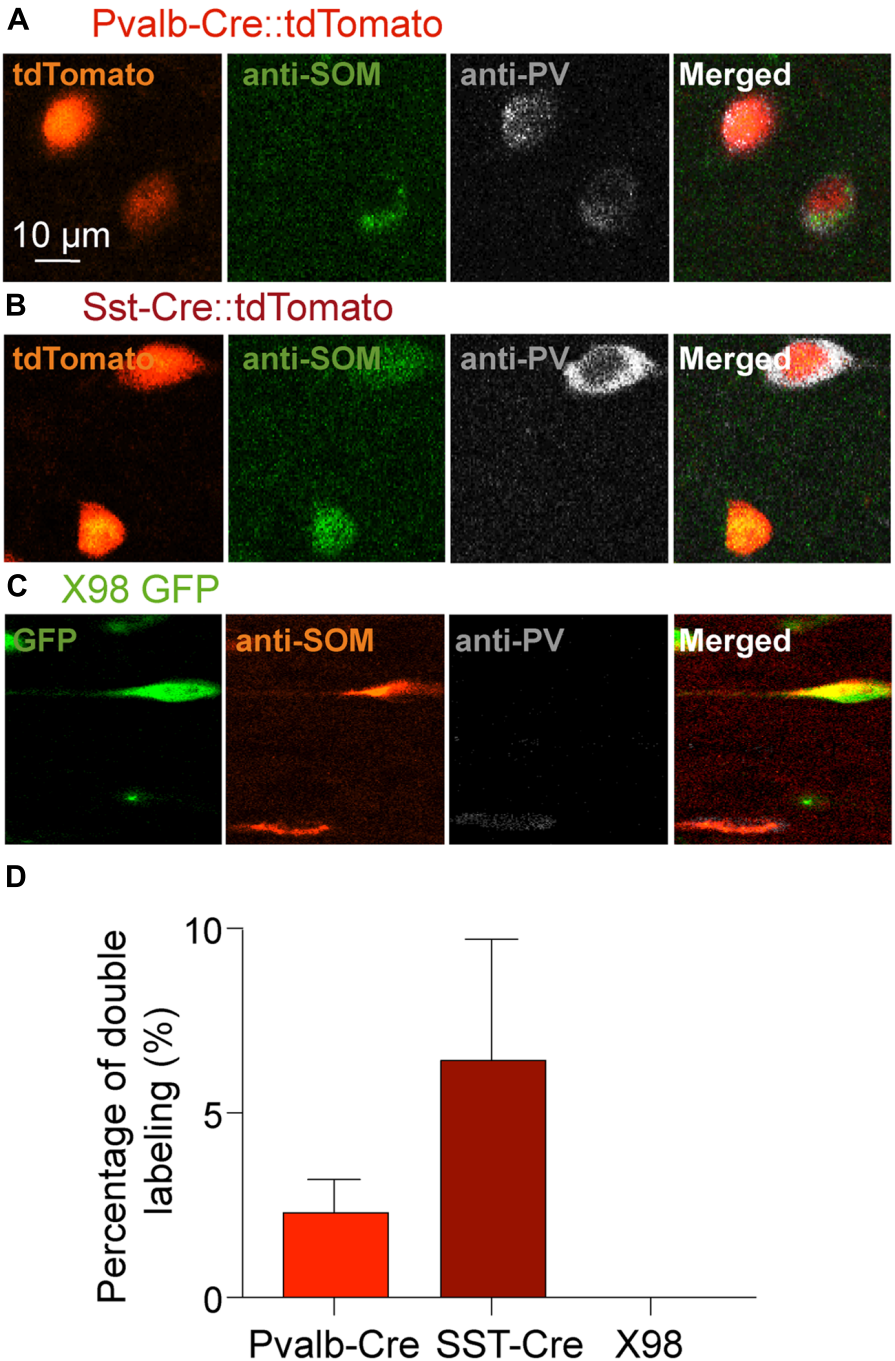
**Double immunostaining of mouse presubicular sections for PV and SOM**. Staining of tissue from **(A)** Pvalb-Cre tdTomato, **(B)** Sst-Cre tdTomato and **(C)** X98 GFP animals with antibodies against SOM (green or red) and PV (gray). **(A–C)** show fluorescent signals from labeled neurons of each mouse line (left), immunostaining for SOM, for PV and a merged image (right). **(D)** 2% of Pvalb-Cre tdTomato+ cells were immunopositive for both PV *and* SOM. 6% of Sst-Cre tdTomato+ cells were positive for both PV *and* SOM. Co-marked cells were found in both deep and superficial layers. Since X98 GFP+ cells were never immunopositive for PV, there was no co-labeling.

We next asked if PV labeled neurons of the Sst-Cre mouse line might reflect the off-target recombination that has been described in this Cre line (Hu et al., 2013), or whether some presubicular interneurons truly express both PV and SOM. **Figure 4** shows the results of double-labeling with anti-PV and anti-SOM antibodies in slices from Pvalb-Cre and from Sst-Cre tdTomato mice. We confirmed that a small proportion of interneurons were immuno-positive for both markers. In the Pvalb-Cre line 2 ± 1% of labeled cells were stained by both antibodies, and in Sst-Cre mice 6 ± 3% of labeled neurons expressed both PV and SOM. This fully accounts for the SOM expressing cells detected in the Pvalb-Cre line. However, neurons co-expressing both PV and SOM only partly account for the numbers of PV expressing neurons detected in Sst-Cre tdTomato+ labeled cells.

Furthermore a number of tdTomato+ cells were not immunopositive for either PV or SOM. Such neither SOM nor PV expressing somata were detected in 7% of Pvalb-Cre tdTomato+ cells (12/181), more frequently in superficial than in deep layers (8 vs. 4% respectively). In the Sst-Cre mice, 19% (85/449) of tdTomato+ cell bodies were not immunopositive for either SOM or PV. These double immunonegative neurons were more numerous in deep (22%) than in superficial layers (14%).

### Cluster Analysis of Electrophysiological Parameters Reveals Groups of Presubicular Interneurons

We measured passive membrane properties, AP waveforms and firing patterns of 142 presubicular interneurons for a classification based on electrophysiological criteria alone. All recordings were made from labeled neurons in slices from the mid-to-dorsal portion of presubiculum. Dorso-ventral depths ranged from -3 to -2.2 mm, with most interneurons recorded from a level close to -2.5 mm (cf. **Figure 1A**). Fluorescent neurons, 46 from Pvalb-Cre tdTomato+ mice, 61 from the Sst-Cre tdTomato+ line and 35 GFP+ neurons of the X98 line, were recorded randomly from either superficial or deep layers of presubiculum. The same physiological parameters were measured for 17 superficial layer pyramidal neurons as an external reference. Unsupervised cluster analysis using Ward’s method (Ward, 1963) was based on 17 electrophysiological variables as listed in Table 1.

**Table 1:** Electrophysiological parameters (mean ± SEM) of presubicular pyramidal cells (PC, *n* = 17) and interneurons recorded from the three mouse lines (X98 GFP, 35; Sst-Cre tdTomato, 61; Pvalb-Cre tdTomato, 46). Using these parameters for Ward’s unsupervised cluster analysis permitted separation of clusters 0, 1, 2, and 3. Values (mean ± SEM) for each parameter are given for each cluster in (B).

	PC			X98 GFP			Sst Cre tdTomato			Pvalb Cre tdTomato		
	Mean	SEM	*n*	Mean	SEM	*n*	Mean	SEM	*n*	Mean	SEM	*n*
**(A)**
RMP (mV)	-78	1	17	-54	1	35	-58	1	61	-65	1	46
R_in_ (MΩ)	250	24	17	376	22	35	285	20	61	148	9	46
Time constant (ms)	28	4	17	36	3	35	21	2	61	10	1	46
Sag	1.04	0.00	17	1.25	0.02	35	1.15	0.01	61	1.10	0.01	46
Rheobase (pA)	84	9	17	40	4	35	72	9	61	175	14	46
I-O gain (Hz/nA; MeanInsF)	275	23	17	748	38	35	917	54	61	1015	92	46
I-O gain (Hz/nA; APs/sec)	270	22	17	732	46	35	885	59	61	1065	98	46
MeanInsF (Hz)	33	3	17	33	3	35	74	8	61	247	19	46
Coefficient of variation	0.21	0.02	17	0.28	0.03	35	0.15	0.01	61	0.06	0.00	46
Latency (ms)	27	3	17	21	2	35	15	1	61	13	2	46
Adaptation Index	0.72	0.04	17	0.66	0.02	35	0.86	0.02	61	0.94	0.02	46
Threshold (mV)	-35.5	0.5	17	-38.2	0.4	35	-38.5	0.5	61	-39.6	0.6	46
Width (ms)	0.56	0.02	17	0.27	0.01	35	0.27	0.01	61	0.20	0.01	46
Amplitude (pA)	84	2	17	83	1	35	77	1	61	72	1	46
AHP (mV)	-14.7	0.6	17	-23.8	0.7	35	-23.5	0.5	61	-23.7	0.6	46
Max depol. rate (V.s^-1^)	517	22	17	598	14	35	571	13	61	637	18	46
Min depol. rate (V.s^-1^)	-134	6	17	-355	11	35	-353	14	61	-498	22	46

	**Cluster 0**			**Cluster 1**			**Cluster 2**			**Cluster 3**		
	**Mean**	**SEM**	***n***	**Mean**	**SEM**	***n***	**Mean**	**SEM**	***n***	**Mean**	**SEM**	***n***

**(B)**
RMP (mV)	-78	1	17	-54	1	65	-60	1	48	-70	1	29
R_in_ (MΩ)	250	24	17	374	17	65	189	11	48	137	17	29
Time constant (ms)	28	4	17	32	2	65	11	1	48	13	2	29
Sag	1.04	0.00	17	1.22	0.01	65	1.11	0.01	48	1.09	0.01	29
Rheobase (pA)	84	9	17	40	3	65	113	8	48	202	23	29
I–O gain (Hz/nA; MeanInsF)	275	23	17	778	28	65	762	41	48	1437	131	29
I–O gain (Hz/nA; APs/sec)	270	22	17	746	35	65	747	43	48	1525	132	29
MeanInsF (Hz)	33	3	17	35	2	65	128	7	48	297	27	29
Coefficient of variation	0.21	0.02	17	0.24	0.02	65	0.07	0.00	48	0.08	0.02	29
Latency (ms)	27	3	17	21	1	65	14	2	48	8	2	29
Adaptation Index	0.72	0.04	17	0.70	0.01	65	0.92	0.02	48	1.00	0.02	29
Threshold (mV)	-35.5	0.5	17	-38.4	0.4	65	-37.4	0.5	48	-42.2	0.7	29
Width (ms)	0.56	0.02	17	0.29	0.01	65	0.23	0.00	48	0.18	0.01	29
Amplitude (pA)	84	2	17	82	1	65	76	1	48	69	2	29
AHP (mV)	-14.7	0.6	17	-23.8	0.5	65	-23.8	0.5	48	-22.9	0.8	29
Maximum depolarization rate (V.s^-1^)	517	22	17	567	11	65	627	14	48	623	26	29
Minimum depolarization rate (V.s^-1^)	-134	6	17	-329	9	65	-409	15	48	-547	30	29

**FIGURE 5:**
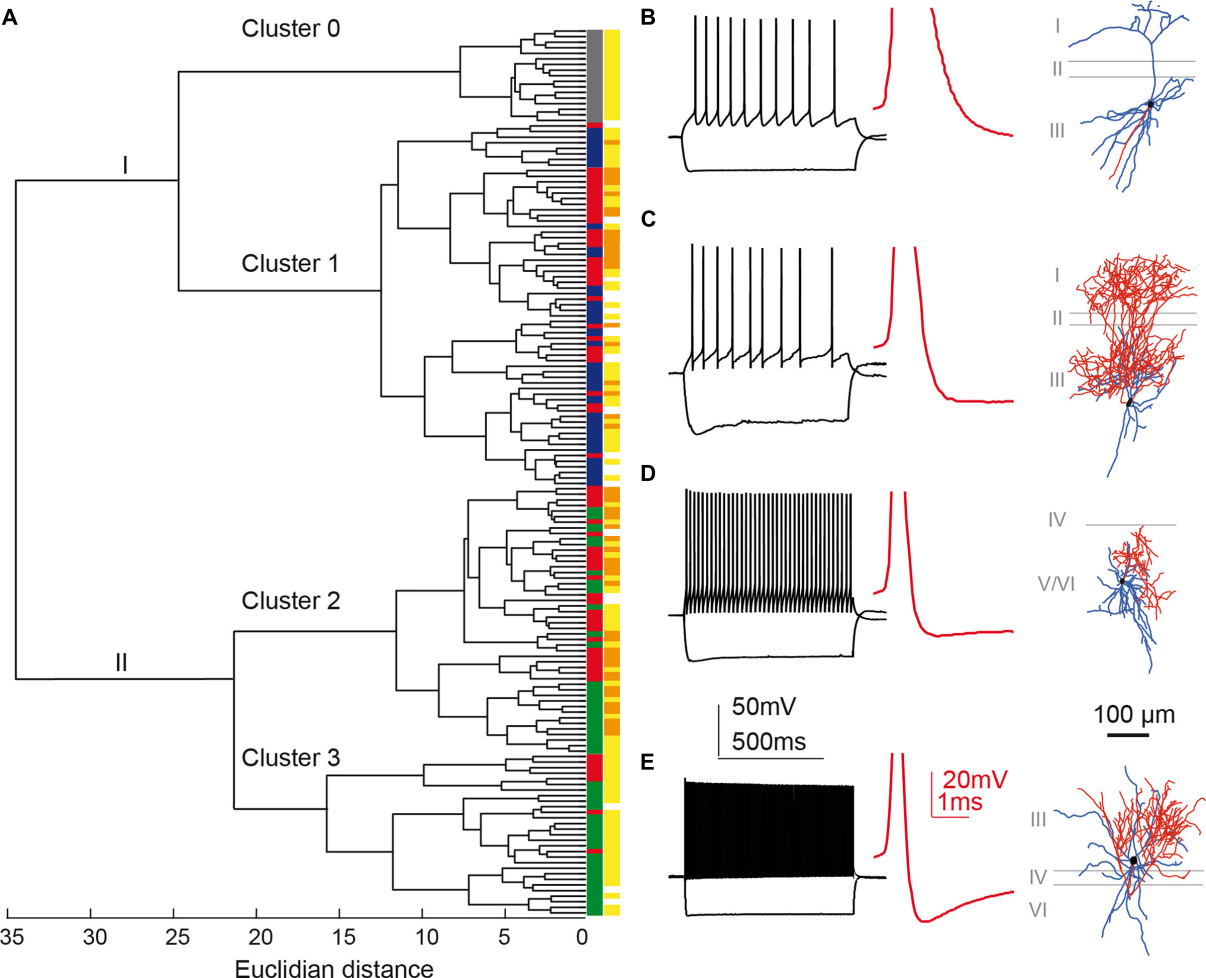
**Cluster analysis classification of presubicular interneurons and pyramidal cells**. Ward’s unsupervised analysis was used with physiological parameters from **Table 1** to classify 17 unlabeled pyramidal cells and 142 fluorescent interneurons from the three mouse lines. **(A)** Dendrogram. Pyramidal cells are isolated in cluster 0 (labeled gray). **(B)** shows a typical pyramidal cell firing pattern and morphology. A cut-off at a Euclidean distance of 20, separated interneurons into clusters 1, 2, and 3. Cluster 1 includes putative SST interneurons from the X98 (blue) and Sst-Cre (red) mouse lines. Cluster 2 includes both Sst-Cre (red) and Pvalb-Cre (green) interneurons. Cluster 3 contains mostly fast-spiking Pvalb-Cre (green) cells. Somatic location was either superficial (yellow) or deep (orange). **(C–E)** show representative neurons from each of these clusters. **(C)**, Martinotti type adapting neuron of cluster 1, recorded from the X98 line. **(D)**, quasi fast-spiking interneuron form cluster 2, recorded from a Sst-Cre tdTomato mouse. **(E)**, fast-spiking interneuron from cluster 3, recorded from a Pvalb-Cre tdTomato mouse. **(B–E)** show current-clamp responses to a negative current pulse that hyperpolarizes the cell to -100 mV and to a positive, twice rheobase current pulse. Insets: details of the first AP repolarization phase (red trace). Morphologies with axons in red and dendrites in blue.

**Figure 5A** shows the hierarchical tree diagram of clusters that emerged. The tree diagram initially separated two populations: the first branch (I) included all principal neurons, all X98 GFP+ cells, and some Sst-Cre tdTomato+ cells; the second branch (II) included all Pvalb-Cre tdTomato+ cells and some Sst-Cre tdTomato+ cells. Decreasing the cut-off value for cluster separation completely isolated the pyramidal neurons, as a highly homogenous population of cells, in cluster 0 (**Figure 5A**). **Figure 5B** shows a typical pyramidal cell. This separation validates the clustering method. While the pyramidal neurons were not considered further, they served as an external reference for the interneuron containing clusters. Three interneuron-containing clusters point to the existence of three main cell types in recorded presubicular interneurons. X98 GFP+ neurons (cluster 1) were strictly segregated from Pvalb-Cre tdTomato+ neurons (clusters 2 and 3). While labeled cells from these two mouse lines were expected to be distributed in distinct clusters, we found a different situation for tdTomato+ interneurons recorded from Sst-Cre mice. A majority of them (55%) clustered together with SOM expressing X98 GFP+ neurons (cluster 1) but a large minority (45%) were grouped together with Pvalb-Cre tdTomato+ interneurons (in clusters 2 and 3). Within-cluster Euclidean distances for clusters 1, 2, and 3 were similar (13, 12, and 15, respectively) even though the Thorndike procedure suggested clusters 2 and 3 might be combined. We explore interneurons in the three clusters in detail below.

### Cluster 1: Mainly Somatostatin Expressing, Low Rheobase Adapting Interneurons

Cluster 1 comprised 65 interneurons, 35 of them GFP+ cells from the X98 mouse line (54%), and 30 Sst-Cre tdTomato+ cells (46%). **Figure 5C** shows a cell of this cluster, a Martinotti type interneuron from the X98 line, with an adapting firing pattern and broad APs. The soma of this SOM expressing, adapting interneuron, typical for cluster 1, was located in layer III.

**FIGURE 6:**
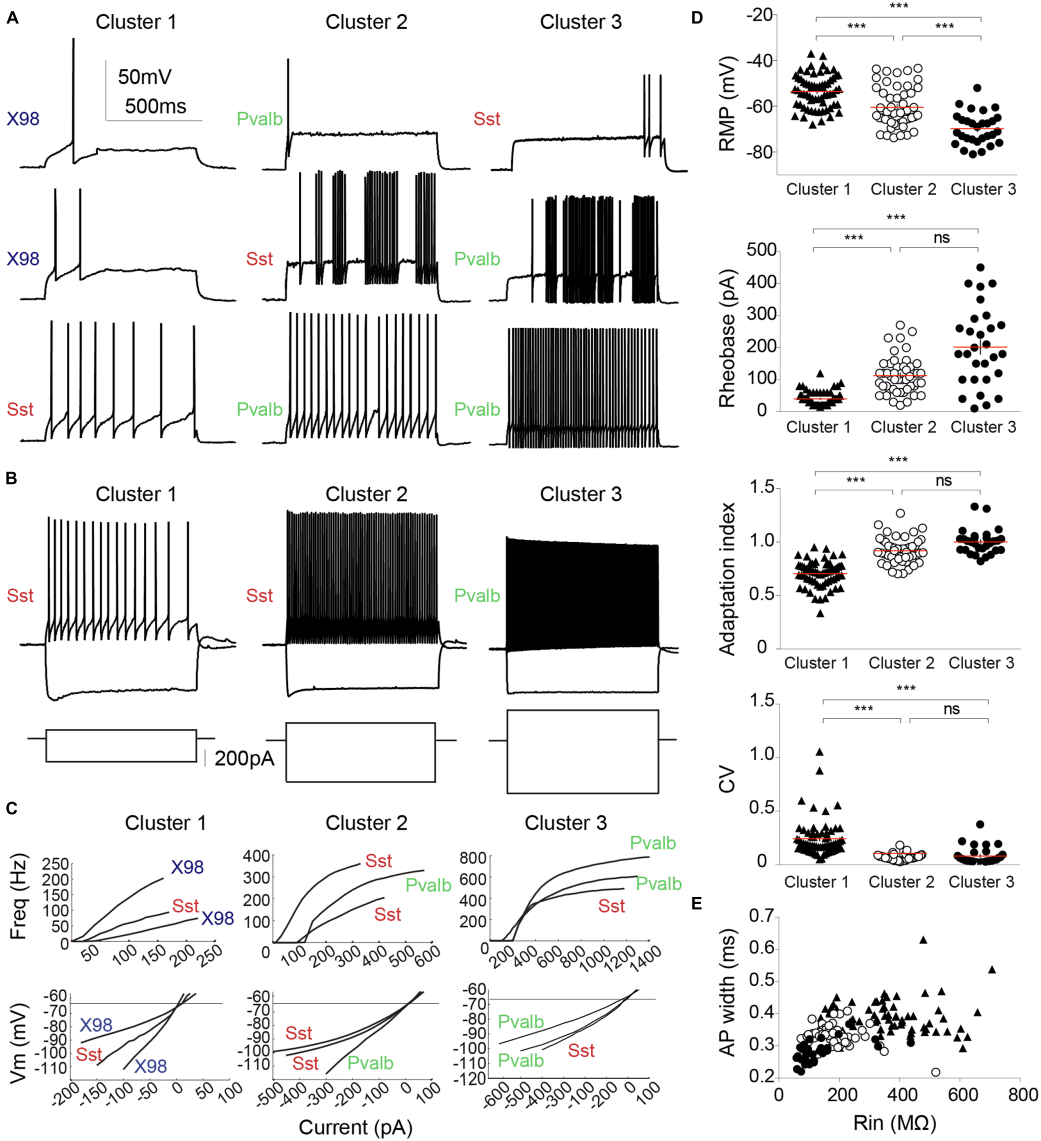
**Electrophysiological diversity of PV or SOM expressing interneurons. (A)** Examples of firing patterns of three different interneurons from each cluster in response to a 800 ms rheobase current pulse. **(B)** Firing induced by a twice rheobase depolarizing current and the trajectory of hyperpolarization to -100 mV induced by a negative current pulse. Note the low R_in_ of neurons in cluster 1 compared with that of neurons in clusters 2 and 3 (larger current steps are needed to elicit similar voltage changes). Most pronounced voltage sags in response to hyperpolarization were exhibited by neurons of cluster 1. **(C)** Input–output curves (upper) and current–voltage relations at sub-threshold potentials (lower) are plotted for three interneurons from each cluster. Left column, Cluster 1; middle, Cluster 2; right column, Cluster 3. Neurons from different mouse lines are identified as: green, Pvalb-Cre tdTomato+; red, Sst-Cre tdTomato+; blue, X98 GFP+. **(D)** RMP, rheobase, AI and CV for each cluster. Each cell is represented by a dot. Horizontal lines (red) indicate mean values. **(E)** AP width plotted against R_in_ for each neuron. Cluster 3 interneurons are characterized by low values for AP width and R_in_. Each cell is represented by a symbol. Cluster 1, filled triangles; Cluster 2, empty circles; Cluster 3, filled circles. Red lines indicate mean values. ^∗∗∗^Kruskal–Wallis and Dunn’s multiple comparison *post hoc* test, ^∗∗∗^*P* < 0.001, ns: non-significant.

Cluster 1 interneurons typically fired spontaneously both in the whole-cell mode and in cell-attached records made before rupturing the membrane. Mean RMP was -54 ± 1 mV (*n* = 65; mean ± SEM), significantly more positive than for the other clusters (*P* < 0.001), and the AP threshold was -38.6 ± 0.4 mV. R_in_ was 374 ± 17 MΩ, twice as high as for cluster 2 cells, and membrane time constant, tau, was 32 ± 2 ms, almost three times longer than in the other two clusters. Hyperpolarizing current injections induced a marked voltage sag (**Figures 6B**; sag ratio 1.22 ± 0.01). Cluster 1 cells could fire regularly or irregularly, with the highest CV at twofold rheobase current levels (0.24 ± 0.02) and a stronger frequency adaptation (AI, 0.70 ± 0.01) than cluster 2 and cluster 3 cells. **Figures 6A,B** (left column) show distinct firing patterns of three cells of cluster 1. Injected currents initiated APs easily, with a mean rheobase of 40 ± 3 pA. Input–output curves, obtained by plotting AP frequency against injected current (**Figure 6C**), had a mean initial slope of 778 ± 28 Hz.nA^-1^. The firing frequency at double rheobase current level was 35 ± 2 Hz, the first AP latency was 21 ± 1 ms. AP mean amplitude was 82 ± 1 mV and width was 0.29 ± 0.01 ms. The maximum AP depolarization and repolarization rates were 567 ± 11 and -329 ± 9 V.s^-1^ respectively. Spike AHPs were sometimes complex or bi-phasic (**Figure 5C**), with mean maximal amplitude of -23.8 ± 0.5 mV.

### Cluster 3: Mostly Fast-Spiking Interneurons from the Pvalb-Cre Line

The 29 neurons of cluster 3 comprised 22 (76%) tdTomato+ neurons of the Pvalb-Cre line, and 7 (24%) from the Sst-Cre line. **Figure 5E** shows a basket shaped interneuron from the Pvalb-Cre line with fast-spiking (FS) firing pattern typical for this cluster. The cell body of this neuron was located in layer III, and all anatomically recovered neurons in this cluster were superficial layer cells.

The mean resting potential of cluster 3 interneurons was -70 ± 1 mV (*n* = 29, mean ± SEM), more negative than values for clusters 1 or 2. Their mean firing threshold was -42.2 ± 0.7 mV, and these cells never fired spontaneously. R_in_ was 137 ± 17 MΩ, half the value of cluster 2 and three times less than cluster 1. The membrane time constant, tau, was 13 ± 2 ms, similar to cluster 2, but almost three times less than for cluster 1. Virtually no voltage sag was observed, even during large hyperpolarizations (**Figure 4B**, right column; sag ratio 1.09 ± 0.01). Induced to discharge by current injection, cluster 3 cells fired with a non-accommodating, FS pattern characteristic of basket cells. **Figures 6A,B** show the stereotyped firing patterns for three neurons from cluster 3 (right column). The CV was low (0.08 ± 0.02) and the AI was 1.00 ± 0.02. The rheobase current to induce firing was 202 ± 23 pA, higher than for clusters 1 or 2. The input–output curves rose steeply, with a high I–O gain of 1437 ± 131 Hz.nA^-1^. Firing frequency at double rheobase current was very high at 297 ± 27 Hz, and AP latency was short (8 ± 2 ms). Single APs of cluster 3 neurons possessed very short half widths (0.18 ± 0.01 ms) with amplitudes of 69 ± 2 mV. Maximum rates of depolarization and repolarization were 623 ± 26 and -547 ± 30 V.s^-1^ respectively. Spike afterhyperpolarization was typically simple with an AHP amplitude of -22.9 ± 0.8 mV.

### Cluster 2: Quasi Fast-Spiking Interneurons from Either Pvalb or Sst-Cre Lines

The 48 neurons of cluster 2 comprised 24 (50%) tdTomato+ neurons of the Pvalb-Cre line, and 24 (50%) from the Sst-Cre line. **Figure 5D** shows a small basket shaped interneuron from the Sst-Cre line with quasi FS properties. The cell body of this neuron was located in layer V/VI, as were many neurons in this cluster.

The membrane potential of cluster 2 cells was -60 ± 1 mV (*n* = 48, mean ± SEM), with a firing threshold of -37.4 ± 0.5 mV. They did not fire spontaneously. R_in_ was 189 ± 11 MΩ and membrane time constant tau was 11 ± 1 ms. The voltage sag upon hyperpolarization was moderate (sag ratio, 1.11 ± 0.01). These values are all intermediate between those of clusters 1 and 3. Cluster 2 neurons fired in regular or quasi-fast patterns (**Figures 6A,B**). CV was low (0.07 ± 0.00), as for cluster 3, and the AI was 0.92 ± 0.02. APs were elicited at a rheobase current of 113 ± 8 pA. Firing gain of mean InsF was 762 ± 41 Hz.nA^-1^, similar to cluster 1 (**Figure 6C**). At double rheobase, the firing frequency was moderate to high (128 ± 7 Hz), and the first AP latency was 14 ± 2 ms. AP amplitude was 76 ± 1 mV and half width 0.23 ± 0.01 ms. Maximal AP depolarization and repolarization rates were 627 ± 14 and -409 ± 15 V.s^-1^ respectively. After-potential waveforms were simple or complex with a mean amplitude of -23.8 ± 0.5 mV.

#### Maternal versus Paternal Inheritance of the cre Transgene

Differential activity of the cre allele may depend on the parent-of-origin (Heffner et al., 2012). We therefore examined the distribution of neurons in each of the three clusters with respect to cre transmission by the male or female parent (Table 2). In the Pvalb-Cre tdTomato mice, cre transmission was paternal for 27 recorded neurons (59%) and maternal for 19 recorded neurons (41%). Cluster 3 with its typical FS cells, contained 22 Pvalb-Cre tdTomato+ neurons, all of which had inherited cre paternally. The intermediate cluster 2 contained 24 Pvalb-Cre tdTomato+ neurons, 20% with cre inherited paternally, and 80% with cre inherited maternally: all Pvalb-Cre tdTomato+ neurons from animals with maternal cre inheritance were grouped in cluster 2, together with five neurons from animals with paternal cre transmission. For the Sst-Cre tdTomato line, cre transmission was paternal for 46 recorded neurons (75%) and maternal for 15 recorded neurons (25%). For the 30 Sst-Cre tdTomato+ neurons of cluster 1, cre transmission was paternal in 87%, and maternal in 13%. For the great majority of the 24 Sst-Cre tdTomato+ neurons in intermediate cluster 2, cre transmission was paternal (79%), and it was maternal in 21%. In cluster 3, the FS cluster, there were seven Sst-Cre tdTomato+ neurons. Only one neuron came from an animal with paternal cre transmission, whereas six of these Sst-Cre tdTomato+ cells in cluster 3 were from animals with maternal cre inheritance (86%).

**Table 2:** Mouse lines and parent-of-origin for Cre lines.

Mouse line and parent-of-origin for Cre lines	Cluster 1	Cluster 2	Cluster 3	Total
X98	35	0	0	35
Sst-Cre paternal	26	19	1	46
Sst-Cre maternal	4	5	6	15
Pvalb-Cre paternal	0	5	22	27
Pvalb-Cre maternal	0	19	0	19

*For each cluster, the number of recorded neurons stemming from each mouse line is given, together with the paternal or maternal cre inheritance for the Sst-Cre and Pvalb-Cre mice*.

#### Morphology

All recorded neurons were filled with biocytin to reveal their anatomy. Axonal and dendritic morphologies of 16 well-filled cells were completely reconstructed with Neurolucida. We compared the morphologies of neurons from all electrophysiologically defined interneuron clusters, and with features of SOM or PV positive interneurons from other cortical areas.

**FIGURE 7:**
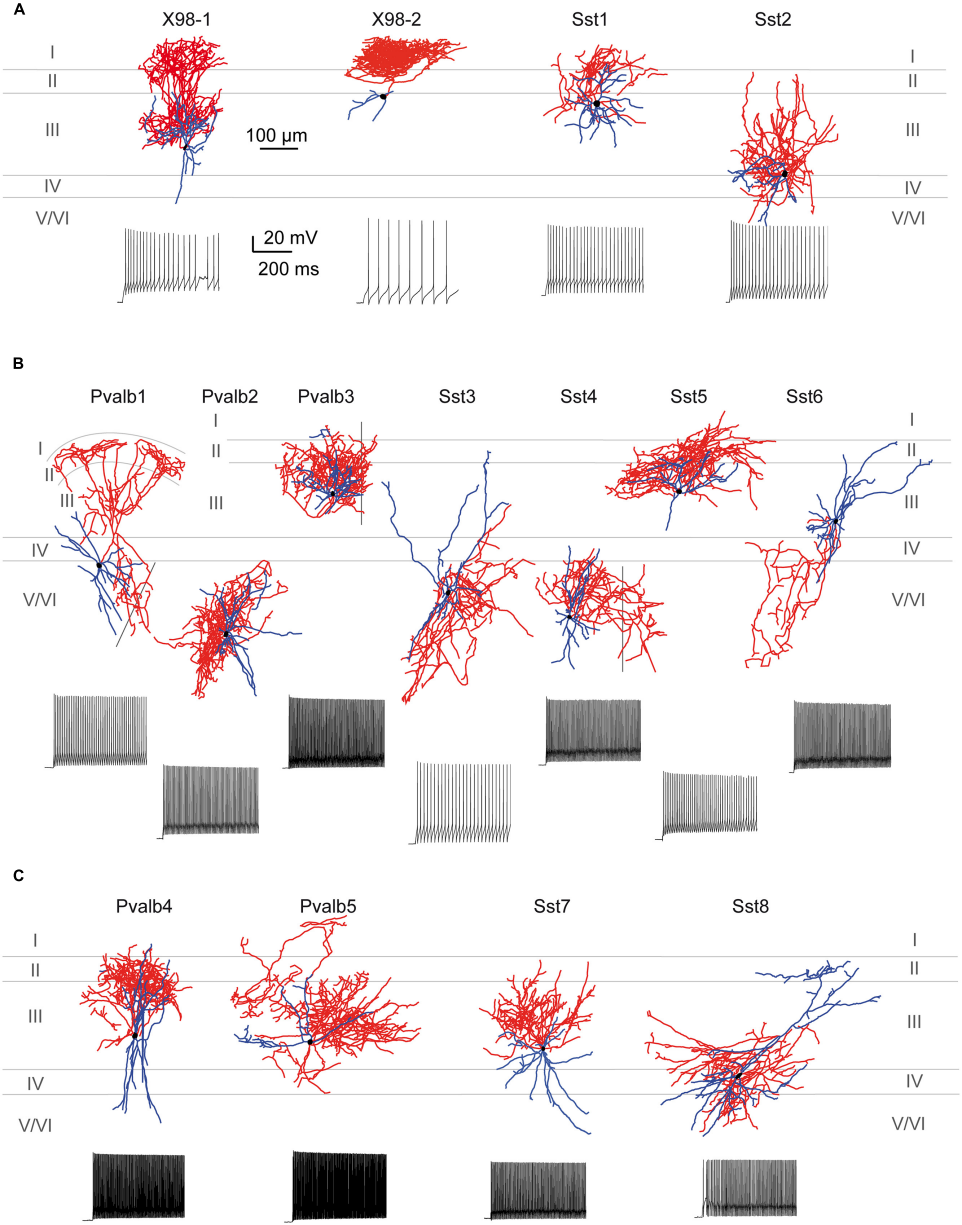
**Morphological variability of presubicular interneurons and firing patterns. (A–C)** Axo-dendritic arbors of 15 biocytin-filled, reconstructed interneurons typical for clusters 1, 2, and 3. Neurons were recorded from the X98, Sst-Cre or Pvalb-Cre mouse lines as indicated. Axons are shown in red, dendrites in blue and cell bodies in black. Firing patterns are shown for each neuron for a double rheobase current injection. **(A)** Cluster 1 comprised putative SOM-expressing interneurons including Martinotti-like (X98-1 and X98-2), GIN-like (Sst1) and X94-like (Sst2) cells. **(B)** Cluster 2 contained interneurons with variable morphology, including basket (Pvalb-1, Pvalb2, Sst3, Sst4) and chandelier-like (Pvalb3) cells as well as GIN-like (Sst5) and atypical SOM-like (Sst6) neurons. **(C)** Cluster 3 contained mainly multipolar basket-cell like interneurons.

**FIGURE 8:**
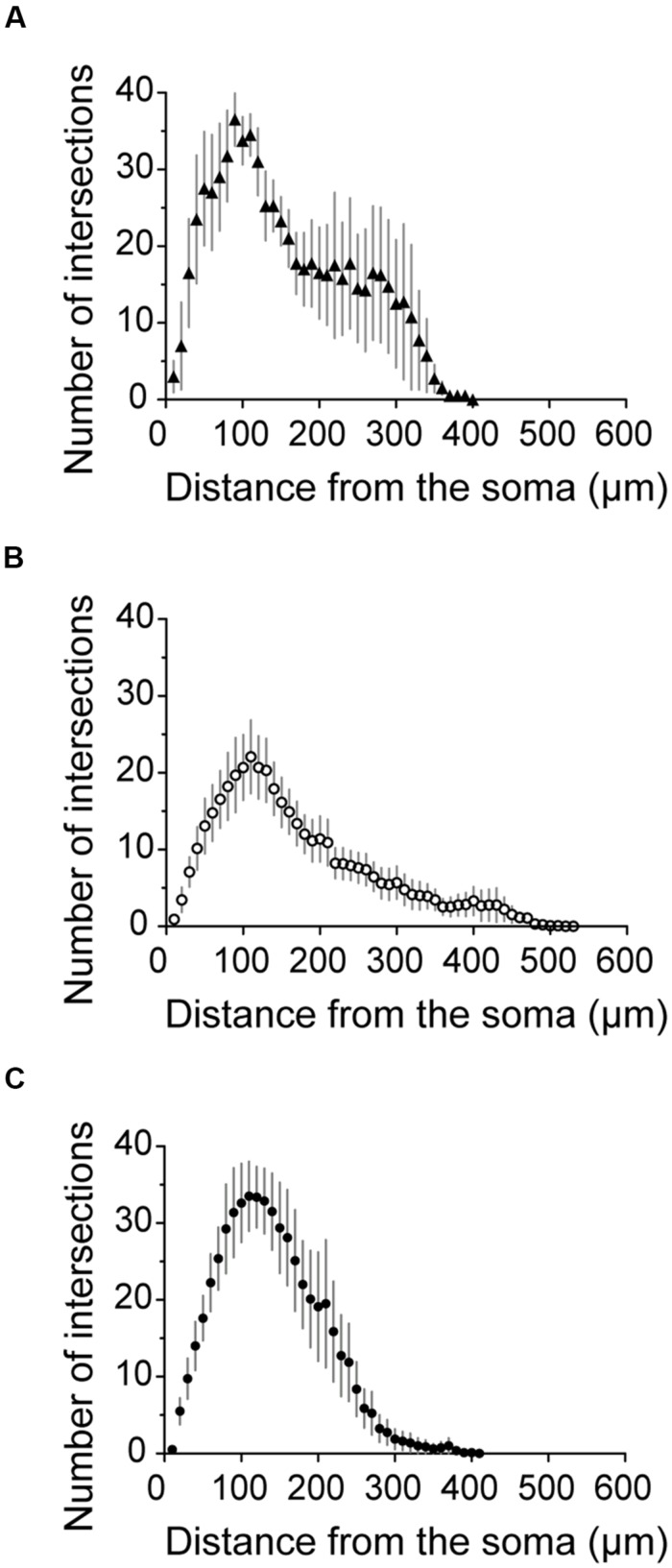
**Axonal Sholl plots. (A–C)** Sholl plots of the number of axon crossings for concentric circles against distance from the soma for neurons from cluster 1 (**A**, filled triangles, *n* = 4), cluster 2 (**B**, empty circles, *n* = 13) and cluster 3 (**C**, filled circles, *n* = 8). Error bars indicate mean ± SEM.

**Figure 7A** shows the somatodendritic form of four cluster 1 neurons. Somata of these putative SOM expressing cells, either X98 GFP or Sst-Cre tdTomato+ labeled, were ovoid, and located in both superficial and deep layers. Axons emerged from the soma or from an ascending dendritic trunk and their arbors were compact. Some branches ramified immediately above the soma in layer III, while multiple collaterals ascended to layer I and branched densely and horizontally for distances as long as 300 μm (Martinotti type interneurons, cf. Wang et al., 2004). Similarly, axons of Sst-Cre tdTomato+ neurons formed a local arbor in the vicinity of the cell body while other axon collaterals projected to, and ramified in layer I (Sst1, “GIN like,” cf. Ma et al., 2006), or, axons avoided layer I (Sst2, “X94 like,” cf. Ma et al., 2006). Axonal Sholl plots accordingly show a complex distribution of axon intersections, with a peak at a distance of 100 μm from the soma, and a plateau between 200 and 300 μm, due to the axonal cluster in layer I (**Figure 8A**). The mean number of primary dendrites for cluster 1 neurons was 4 ± 0.4 (*n* = 4). X98-labeled neurons possessed multipolar dendritic arbors. Sst-Cre tdTomato+ neuron dendrites occupied a radius of 150–200 μm around the soma and were preferentially oriented toward deeper layers. The mean total axonal length of cluster 1 interneurons was 9804 ± 2103 μm, significantly higher than that for cluster 2 and 3 interneurons. A high proportion of the total axon length ramified in layer I (35 ± 12%). The mean axonal length of cluster 1 interneurons was almost 6 times greater than the dendritic length (1764 ± 397 μm; *n* = 4).

**Figure 7B** shows seven completely reconstructed cluster 2 interneurons. Their somata were located either in superficial or deep layers. The mean total axonal length for all interneurons of cluster 2 was 7100 ± 1180 μm (*n* = 13). 6 ± 0.3 primary dendrites projected from the soma of cluster 2 interneurons, usually in all directions for distances up to 500 μm and with a total dendritic length of 2299 ± 160 μm (*n* = 13).

Axonal and dendritic arbors of some superficial cluster 2 neurons, with somata in layers II/III, were entirely limited to superficial layers (see Pvalb3 of axo-axonic like morphology, and Sst5). Reciprocally, axons and dendrites of other neurons with somata in deep layers, were restricted to deep layers, (see Pvalb2, Sst4, and eight other neurons not shown). Axons typically ramified symmetrically around the soma with no specific directional bias as for cortical basket cells. Of the reconstructed neurons shown in **Figure 7B**, some dendrites of cell Sst3 extended away from the somatic layer V to superficial layers II and III. Conversely, the axon of Pvalb1, with a soma in layer V, projected to both deep and superficial layers and ramified especially densely in layer II. Atypically, the axon of Sst6, with soma in layer III, ramified sparsely over large volumes of deep layers almost completely distinct from zones occupied by the dendrites of the neuron. Axons of six cluster 2 interneurons projected into nearby parasubiculum (**Figure 7B**, black vertical lines) where they could ramify extensively (Sst4, axonal length 3063 μm, 45% of total length; Pvalb1, length 2187 μm, 24% of total length) and Pvalb3 (1569 μm, 17% of total length). Only 2 ± 1% of the total axonal length was in layer I. The Sholl analysis for axons of cluster 2 interneurons showed a peak in the number of intersections at a distance of 100 μm, which then decreased gradually to the extremities of the axon at ∼450 μm from the soma (**Figure 8B**).

**Figure 7C** shows four interneurons of cluster 3, with somata all located in superficial layers. The mean total axonal length of reconstructed cluster 3 interneurons was 8511 ± 1488 μm and mean total dendritic length 2370 ± 344 μm (*n* = 8). Axons were typically restricted to superficial layers and collaterals tended to ascend rather than descend (Pvalb4, Pvalb5, and Sst7). Axonal arbors could be dense, such as that of Pvalb5 which projected long axon collaterals in all directions, as do large basket cells (Wang et al., 2002). Sholl analysis of axon distributions showed a peak at 100 μm from the soma, and axons typically did not project more than 300 μm from the soma (**Figure 8C**). There were 6 ± 0.6 primary dendrites in cluster 3 interneurons. Dendritic arbors were typically multipolar, ramifying evenly in all directions from the soma, as for neurons of cluster 2. Other dendritic arbors such as that of Pvalb4 (and one similar neuron not shown) possessed vertically biased dendrites projecting to both deep and superficial layers or just toward superficial layers (Pvalb5) or deep layers (Sst8). Similar to cluster 2, cluster 3 was characterized by a low proportion of the total axonal length in layer I (1 ± 1%).

## Discussion

This study provides a first classification of mouse presubicular interneurons. Our data show about 11% of mouse presubicular neurons are GABAergic. We used transgenic animals to identify subtypes of these cells that express the peptide SOM or the Ca-binding protein PV. We measured physiological parameters and somato-dendritic form and also verified the chemical content of labeled neurons from Pvalb-Cre, Sst-Cre and X98 mice. Immunohistochemical verification of these interneuron markers revealed both a true overlap – about 5% of presubicular interneurons were co-labeled by antibodies against SOM and PV – and an imperfect specificity for one of the animal lines – only about 70% of labeled cells in Sst-Cre animals were immunopositive for SOM. We therefore used physiological parameters alone for a cluster analysis. Three groups of interneurons emerged. Cells conforming to classical archetypes of adapting SOM neurons and FS PV neurons occupied two distinct clusters (1 and 3). A third cluster (cluster 2) contained quasi fast spiking neurons with intermediate properties. Neurons from the cluster of FS cells (3) often possessed a basket-cell like anatomy, those from the cluster of neurons with adapting firing (1) could display a Martinotti cell like anatomy, but neurons with intermediate physiology (2) tended to possess intermediate somato-dendritic forms.

### Mouse Lines and Neurochemical Marker Expression Pattern in Presubiculum

Labeled cells from genetically modified mouse lines revealed a specific distribution of interneuron subtypes in distinct layers of presubiculum, with the exceptions for mislabeling. The overall density of GABAergic interneurons in different layers was measured using the GAD67-GFP line. Pvalb-Cre tdTomato+ labeled neurons were preferentially located in superficial layers (II/III) innervated by thalamic afferents while the density of Sst-Cre tdTomato+ cells was highest in deeper layers (V/VI) of the presubiculum. Layer specific distributions of distinct types of interneurons may differ in other cortical regions. In mouse visual cortex, PV, and SOM interneurons are more evenly distributed across superficial and deep layers (Gonchar et al., 2007). However, higher SOM positive cell densities in deep cortical layers, as here, were previously described by Ma et al. (2006) and Xu et al. (2010). In contrast to our results, PV cell density has been found to be higher in deep than in superficial cortical layers (Xu et al., 2010). There may be differences between visual, frontal and somatosensory cortical areas, as well as differences between mouse and rat cortex (Ma et al., 2006; Xu et al., 2010).

Immunohistochemistry showed that ∼90% of labeled cells from Pvalb-Cre animals were positive for PV and ∼2% were SOM positive. Double immunolabeling demonstrated a co-expression of SOM *and* PV. Even though PV and SOM expression do not overlap in neocortical adult interneurons of rodents (Gonchar and Burkhalter, 1997; Kawaguchi and Kubota, 1997; Xu et al., 2010; Kubota et al., 2011), mRNAs coding for both PV and SOM have been detected in the same interneuron (Cauli et al., 2000). PV and SOM co-expression has been detected by immuno-labeling of bistratified neurons or oriens-locunosum-moleculare interneurons of the hippocampus (Jinno and Kosaka, 2000; Klausberger et al., 2003; Somogyi and Klausberger, 2005; Fishell and Rudy, 2011; Katona et al., 2014).

Immunostaining showed that ∼70% of labeled Sst-Cre tdTomato+ cells were SOM positive, while 23% were PV positive. Cre-mediated recombination in this mouse line had been assumed to be largely restricted to SOM interneurons (Gentet et al., 2012; Cottam et al., 2013; Kvitsiani et al., 2013; Xu et al., 2013). Data of Hu et al. (2013) suggest though that 6–10% of labeled interneurons in different cortical areas of Sst-Cre tdTomato+ mice possess a fast spiking PV-like phenotype. This result may be explained by off-target recombination in PV cells that transiently express SOM during development (Hu et al., 2013), possibly at high levels in the presubiculum. Off-target recombination may occur preferentially for maternal cre inheritance. However, PV and SOM are co-expressed in 6% of Sst-Cre tdTomato+ neurons. This special population of PV+SOM+ cells does not entirely account for all (23%) PV labeled Sst-Cre tdTomato+ neurons. The 16% of Sst-Cre neurons that expressed neither SOM nor PV, could correspond to other types of interneurons. Some Sst-Cre tdTomato+ cells could be labeled with anti Calbindin antibody for instance (data not shown). Alternatively they might be non-GABAergic which could explain how the Sst-Cre tdTomato+ neuron density exceeded that of GAD67-GFP neurons in layers V/VI. Indeed we also noted some clusters of pyramidal shaped Sst-Cre tdTomato+ neurons in adjacent subiculum (unpublished observation).

The X98 mouse line is specific for a subset of infragranular, SOM containing interneurons in neocortex (Ma et al., 2006). In the presubiculum, ∼90% of labeled cells were immunopositive for SOM and 0% for PV. The somata of labeled cells from X98 mice were located in both deep and superficial layers, with a highest density in layer IV.

### Interneuron Classification

Classical anatomical studies of Cajal (1911) and Lorente de No (1933) established that short-axon cells possess diverse forms. More recently, interneurons have been classified into distinct groups on the basis of electrophysiological, morphological, molecular and developmental criteria (Markram et al., 2004; Petilla Interneuron Nomenclature Group et al., 2008; Druckmann et al., 2012). But the fundamental question of how to define different classes of interneurons (Parra et al., 1998) or how to treat a structured continuum (Battaglia et al., 2013) remains open. Here we used Ward’s unsupervised classification method to analyze interneuron types based on multiple physiological parameters. The resulting groups were then correlated with the maternal versus paternal inheritance of the cre transgene and the neuronal anatomy.

Ward’s method requires no preliminary supposition on the number of cell types, even if it may separate neuronal classes less efficiently than K-mean clustering (Cauli et al., 2000; Karagiannis et al., 2009). We included unlabeled presubicular pyramidal cells as a control of our cluster analysis. Their clear separation from all labeled interneurons, as cluster 0, validates our clustering procedures. Application of the Thorndike procedure to Ward’s clustering method should provide an optimal threshold to maximize information content. With this threshold, adapting SOM interneurons (**Figure 5C**) labeled from X98 mice were found in cluster 1, while classical FS interneurons (**Figure 5E**) from the Pvalb-Cre line were grouped in main branch II of the dendrogram. These two interneuron classes have been defined in both hippocampus and neocortical areas (Cauli et al., 1997; Kawaguchi and Kubota, 1997; Markram et al., 2004; Somogyi and Klausberger, 2005; Petilla Interneuron Nomenclature Group et al., 2008; Fishell and Rudy, 2011). Lowering the cluster separation threshold permitted resolution of two distinct clusters 2 and 3. Interneurons of cluster 2 possessed intermediate, physiological and anatomical properties. We were surprised to note that interneurons labeled in the Sst-Cre line were found in all interneuron containing clusters (cluster 1, 2, and 3).

### Two Main Types of GABAergic Interneurons in Presubiculum

Cluster 1 interneurons fired regularly with a consistent frequency adaptation. APs were characterized by a large amplitude and half-duration. These cells were the most excitable of the three groups with depolarized membrane potentials, high R_ins_ and pronounced voltage sags. All cells in this cluster were from SOM expressing X98 interneurons or from the Sst-Cre line, in majority with paternal cre inheritance. Similar adapting-SOM type interneurons are described in hippocampus as oriens-lacunosum-moleculare cells and in neocortex as Martinotti cells (Wang et al., 2004; Halabisky et al., 2006; Ma et al., 2006; Uematsu et al., 2007; Karagiannis et al., 2009; Xu et al., 2013).

Anatomically, X98 GFP+ labeled cells of cluster 1 resembled Martinotti cells. They possessed multipolar dendrites, with few primary dendrites as for cortical SOM Martinotti cells (Kawaguchi et al., 2006). Axons sent collaterals to layers II/III and particularly to layer I, where they could branch over horizontal distances up to 300 μm (Wang et al., 2004; Ma et al., 2006). Axonal arborizations of Sst-Cre tdTomato+ labeled neurons were more sparse and ramified locally around their soma, as do some SOM neurons of the GIN mouse line (McGarry et al., 2010). Cluster 1 interneurons are well suited to control inputs from retrosplenial cortex and thalamus which excite principal cell apical dendrites in presubicular layers I and III (van Groen and Wyss, 1990b; Kononenko and Witter, 2012).

Cluster 3 comprised FS cells, similar to those of hippocampus (Somogyi and Klausberger, 2005) and neocortex (Kawaguchi, 1995). Neurons of this group were the least excitable in responses to current injection. They possessed hyperpolarized resting potentials, low R_in_ and short membrane time constants (**Figure 6**). Stronger stimuli induced sustained high-frequency firing of fast spikes with little or no frequency adaptation, linked to fast, delayed rectifier Kv3-mediated currents (Martina et al., 1998; Erisir et al., 1999). Hence, the AP firing pattern can be described as fast spiking, with continuous delayed or stuttering dynamics (Druckmann et al., 2012).

Most interneurons of cluster 3 were recorded from Pvalb-Cre animals, with paternal cre inheritance only, and others from the Sst-Cre line with mostly maternal inheritance (Table 2). Possibly the second group corresponds to off-target recombination (**Figure 3B**). Anatomically, archetypal FS-PV interneurons include basket cells and chandelier cells of neocortical superficial layers (Kawaguchi, 1995; Wang et al., 2002) and of the hippocampus (Freund and Buzsáki, 1996; Somogyi and Klausberger, 2005). Dendritic arbors of filled neurons of cluster 3 FS cells were typically multipolar, with more primary dendrites than SOM cells of cluster 1 (cf. Kawaguchi et al., 2006). Axonal distributions conformed to those of these cell types although we did not confirm a perisomatic site of postsynaptic targets. Axonal arbors could be small or large and while some projections remained local, others might mediate a translaminar or transcolumnar inhibition (Wang et al., 2002; Karube et al., 2004; Markram et al., 2004). The tdTomato+ cells of the Sst-Cre line in this cluster had basket like morphologies similar to the Pvalb-Cre tdTomato+ neurons.

### … and An Intermediate Cluster

Cluster 2 grouped together some Sst-Cre tdTomato+ interneurons with similar numbers of Pvalb-Cre tdTomato+ interneurons (**Figure 5A**). *Cre* inheritance could be either paternal or maternal, but all Pvalb-Cre cells from animals that inherited cre maternally were found solely in this cluster. Electrically, neurons of this cluster possessed intermediate values of membrane potential, a relatively small resistance and a short time constant approaching that of FS cells. The APs of cluster 2 cells from both Cre mouse lines were of short duration and firing patterns included single spiking, stuttering and regular spiking (**Figure 6**). At higher firing frequencies, neurons of this cluster displayed a quasi-FS firing pattern with a weak to absent frequency adaptation. Cluster 2 cells in presubiculum are thus clearly distinct from the classical adapting SOM-archetype of cluster 1. In other neocortical areas, SOM positive neurons comprise several subtypes, including the Martinotti type cells (Wang et al., 2004), the SOM cells in the X94 line (Ma et al., 2006), and others (Halabisky et al., 2006; McGarry et al., 2010). It is possible that the subpopulation of interneurons co-expressing SOM and PV (**Figure 4**) were included in cluster 2, even though with restricted numbers, they seem unlikely to account for all of the cells.

The somato-dendritic morphology of labeled neurons from both Sst-Cre and the Pvalb-Cre lines grouped into cluster 2 was often similar to that of basket cells (Wonders and Anderson, 2006; Kubota, 2014). Neurites of these cells tended to branch within their home layer with also intra-laminar axonal projections that may mediate early and late blanket inhibition (Karnani et al., 2014). Other neurons of cluster 2 did not follow this pattern, with dendrites oriented toward superficial (layer II/III) or deep layers (V/VI). Conversely, other neurons with dendritic arbors in superficial layers projected axons into deep layers, and presumably mediate translaminar inhibition (Bortone et al., 2014).

### Interneuron Diversity

Presubicular interneurons examined here included both typical SOM adapting cells and classical FS PV cells. However, we also distinguished a group of interneurons with intermediate physiology and anatomy. Labeled neurons of this cluster (2) comprised all Pvalb-Cre tdTomato+ neurons with maternal cre inheritance, some with paternal inheritance, as well as Sst-Cre tdTomato+ neurons with either paternal or maternal cre inheritance. These interneurons seem to represent a convergence of traits of archetypical SOM- and PV-containing cells. Such a continuum of properties has been evoked in the context of interneuron classification and linked to fuzzy set theory (Battaglia et al., 2013).

How might cells with intermediate traits emerge? Interneuron properties are specified during development. Both PV and SOM interneurons, along with the majority of neocortical interneurons, originate from the medial ganglionic eminence (Xu et al., 2004; Wonders and Anderson, 2006; Batista-Brito and Fishell, 2009; Miyoshi et al., 2010; Kepecs and Fishell, 2014). While adapting-SOM and FS PV cells may be archetypal, a common developmental origin might also produce transitional “edge cells” reflecting a shared embryonic origin. Clones of the same progenitor lineage include both SOM- and PV-expressing interneurons rather than a single subtype (Kepecs and Fishell, 2014). Could that explain the presence of Sst-Cre and Pvalb-Cre tdTomato+ cells in a same interneuron class? Even transient SOM expression in Sst-Cre cells should induce a persistent tdTomato+ signal due to Cre-recombinase expression and Cre-mediated recombination (Hu et al., 2013). This point should be pursued by a molecular characterization, including calcium binding protein and neuropeptide content, of Sst-Cre tdTomato+ interneurons with quasi-FS properties. Further studies on parent-of-origin effect for *cre* transmission should examine why different interneuron phenotypes are labeled or whether maternal cre inheritance could modify interneuron phenotype.

Parvalbumin and somatostatin expressing neurons both originate in the MGE. They are presumably subject to similar chemical cues during migration and when they arrive in the presubiculum similar local cues control interneuron phenotype and neurite branching pattern (Adams and Eichmann, 2010; Battaglia et al., 2013). Even if the six-layered cytoarchitecture of the presubiculum is similar to that of neocortex, the heterogeneous, atypical populations of presubicular PV and SOM interneurons may be linked to the transitional nature of the region (O’Mara et al., 2001; Simonnet et al., 2013). Possibly similar features of Sst-Cre and Pvalb-Cre tdTomato+ cells in cluster 2 originate from local presubicular signals.

## Implication of Interneurons in Presubicular Microcircuit Function

GABAergic neurons of the presubiculum seem likely to control the timing, sensitivity and selectivity of head directional signals. Synapses of FS basket-like cells of cluster 3 presumably target perisomatic regions of principal cells and act to enforce precisely timed firing as in hippocampus or somatosensory cortex (Miles et al., 1996; Fricker and Miles, 2000; Pouille and Scanziani, 2001; Gabernet et al., 2005). In contrast, inhibition due to Martinotti cells of cluster 1, targets dendritic sites in layer I and may act to counter layer-specific excitatory inputs from retrosplenial cortex and thalamus in a graded fashion. Martinotti cells are often reciprocally connected with presubicular pyramidal cells (unpublished observations), and their recruitment should tend to limit excitatory inputs or mediate lateral inhibition onto nearby cells (Silberberg and Markram, 2007). Mapping by two-photon glutamate uncaging showed that inhibition by both PV and SOM cortical interneurons induces a “blanket” of inhibitory actions rather than locally selective effects (Fino and Yuste, 2011; Packer and Yuste, 2011). Presubicular PV or SOM interneurons with sparse or profuse local axonal arborisations seem likely also to target postsynaptic targets non-selectively and spread a “blanket of inhibition” over the microcircuit (Karnani et al., 2014). Dual records from interneurons and principal cells will be needed to establish connectivity motifs of distinct groups of presubicular interneurons for comparison with inhibitory circuits in other regions of the cortex.

## Conflict of Interest Statement

The authors declare that the research was conducted in the absence of any commercial or financial relationships that could be construed as a potential conflict of interest.

## Abbreviations

AHP: after-hyperpolarization
AP: action potential
GFP: green fluorescent protein
InsF: instantaneous frequency
PV: parvalbumin
R_in_: input resistance
RMP: resting membrane potential
SOM: somatostatin
